# Recent advances in cellular biosensor technology to investigate tau oligomerization

**DOI:** 10.1002/btm2.10231

**Published:** 2021-06-01

**Authors:** Chih Hung Lo

**Affiliations:** ^1^ Department of Neurology, Brigham and Women's Hospital Harvard Medical School Boston Massachusetts USA

**Keywords:** Alzheimer's disease (AD), bimolecular fluorescence complementation (BiFC), cell‐based biosensor, conformational ensembles, fluorescence resonance energy transfer (FRET), high‐throughput screening (HTS), protein–protein interaction (PPI), split fluorescent protein complementation, split luciferase complementation (SLC), tau oligomerization

## Abstract

Tau is a microtubule binding protein which plays an important role in physiological functions but it is also involved in the pathogenesis of Alzheimer's disease and related tauopathies. While insoluble and β‐sheet containing tau neurofibrillary tangles have been the histopathological hallmark of these diseases, recent studies suggest that soluble tau oligomers, which are formed prior to fibrils, are the primary toxic species. Substantial efforts have been made to generate tau oligomers using purified recombinant protein strategies to study oligomer conformations as well as their toxicity. However, no specific toxic tau species has been identified to date, potentially due to the lack of cellular environment. Hence, there is a need for cell‐based models for direct monitoring of tau oligomerization and aggregation. This review will summarize the recent advances in the cellular biosensor technology, with a focus on fluorescence resonance energy transfer, bimolecular fluorescence complementation, and split luciferase complementation approaches, to monitor formation of tau oligomers and aggregates in living cells. We will discuss the applications of the cellular biosensors in examining the heterogeneous tau conformational ensembles and factors affecting tau self‐assembly, as well as detecting cell‐to‐cell propagation of tau pathology. We will also compare the advantages and limitations of each type of tau biosensors, and highlight their translational applications in biomarker development and therapeutic discovery.

## 1 INTRODUCTION

**FIGURE 1:**
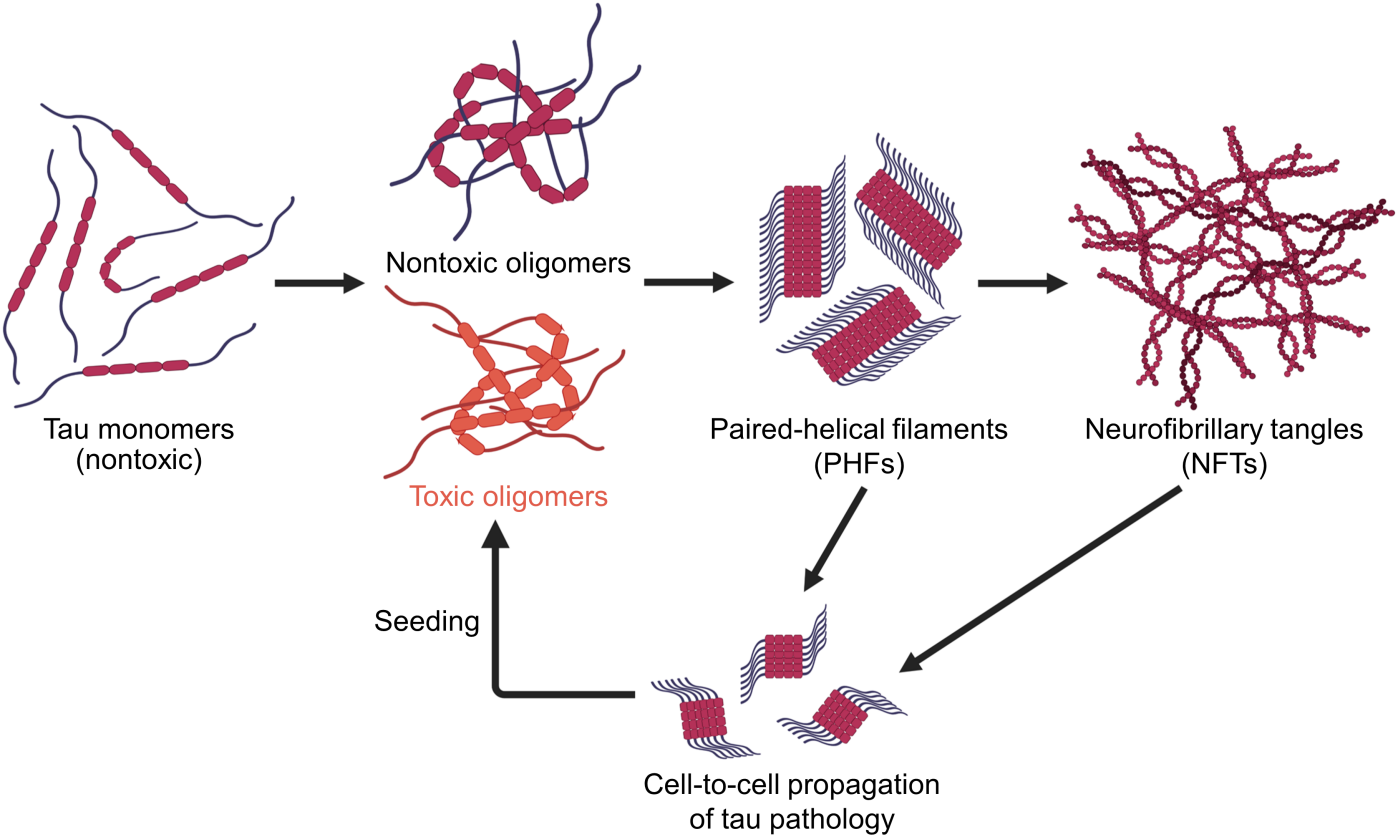
Tau fibrillogenesis cascade and cell‐to‐cell propagation of tau pathology in Alzheimer's disease. Misfolded tau species is capable of forming both nontoxic and toxic soluble tau oligomers spontaneously. The tau oligomers can proceed to form paired helical filaments (PHFs) and neurofibrillary tangles (NFTs) which are large insoluble aggregates with β‐sheet conformations. The fibrillar species can be secreted by host cells and transmitted to recipient cells which is capable of inducing further seeded oligomerization or aggregation, leading to cell‐to‐cell propagation of tau pathology. Schematics are created with BioRender.com

Alzheimer's disease (AD) is the sixth leading cause of death for all adults in the United States and one of the top three fatal diseases for older people over the age of 65.^1^ It is the most common form of dementia and afflicting more than 25 million people in the world, but there is currently no effective disease‐modifying therapy.^2^, ^3^ AD is a neurodegenerative disease and an example of tauopathies with a marked increase in the number of tau inclusions such as neurofibrillary tangles (NFTs) in affected brain regions of dementia patients.^4^ Tau exists as an intrinsically disordered monomer that plays a crucial role in the regulation of signaling pathways, axonal stability and microtubule stability by binding multiple different molecules.^5^, ^6^ Under pathological conditions such as abnormal posttranslational modifications, pathogenic mutations or hyperphosphorylation, tau misfolds with conformational changes, accumulates in the cytosol and initiates the fibrillogenesis cascade.^4^ The aggregation pathway initiates with the spontaneous formation of tau oligomers from monomers and subsequently nucleates into paired helical filaments, and eventually intracellular NFTs (Figure 1). While the large insoluble NFTs have been the histopathological hallmark of AD and tauopathies, the soluble tau oligomers that are formed prior to fibril formation has been proposed to be the principal toxic species in recent studies.^7^, ^8^ These toxic tau oligomers promote cellular cytotoxicity^9^, ^10^, ^11^ and induce cognitive deficits and neurodegeneration in animal models.^12^, ^13^, ^14^, ^15^ As a result, there is a shift in the therapeutic paradigm to inhibit or disrupt the formation of toxic tau oligomers, rather than the large insoluble fibrillar aggregates.^16^, ^17^, ^18^, ^19^

Tau oligomers exist as a heterogeneous ensemble of distinct assemblies with molecular diversity including both fibrillization competent and resistant, toxic, and nontoxic species.^20^, ^21^, ^22^ Toxic tau oligomer contributes to tau pathology by initiating tau aggregation, inducing toxicity and propagating tau species through cell‐to‐cell spreading (Figure 1).^23^ Toxicity arises from cellular dysfunctions such as apoptosis induced by activated caspase and mitochondrial impairments, which impede synaptic energy production and lead to neuronal death.^24^, ^25^ The cell‐to‐cell spreading phenomenon is characterized by the ability of cells to secrete and uptake tau in the naked form^26^, ^27^ or through other vesicles such as exosomes.^28^ In terms of tau aggregation, it is important to note that wild‐type (WT) full‐length tau is resistant to fibrillization and forms mostly soluble oligomers.^29^, ^30^, ^31^ On the other hand, tau mutants and truncated forms of tau are more prone to oligomerization, with a higher tendency of forming insoluble tau aggregates or fibrils.^32^

The most promising approach to target toxic tau oligomers will be to take advantage of the available knowledge on the conformational ensembles or structures of these species.^33^, ^34^ A substantial number of studies have made use of purified recombinant tau oligomers that are assembled in vitro to explore their biophysical properties (e.g., conformational changes and protein–protein interactions [PPIs]) and toxicity.^9^, ^11^, ^35^, ^36^, ^37^, ^38^, ^39^, ^40^, ^41^, ^42^, ^43^, ^44^ While several studies have shown that WT full‐length tau forms dimers and trimers spontaneously due to disulfide bond formation,^35^, ^36^, ^38^, ^42^, ^43^, ^44^ others have generated self‐assembled tau oligomers in the presence of aggregation‐prone mutations or truncations, or with the help of aggregation inducers such as heparin or tau seeds.^9^, ^11^, ^37^, ^39^, ^40^, ^41^ However, it is often difficult to control the extent of aggregation to obtain toxic oligomers with these inducers as fibrils or a mixture of oligomers and fibrils may be formed, which will interfere with the investigations of tau oligomers. In addition, established protocols to generate tau oligomers and aggregates from purified proteins have been shown to produce different tau assemblies depending on aggregation conditions, without specific toxic tau species being identified.^45^ Furthermore, it is important to note that although some of these purified tau oligomers are capable of inducing toxicity, they lack numerous chaperone proteins present in cells, and hence do not recapitulate tau oligomerization in the cellular environment.^46^

**FIGURE 2:**
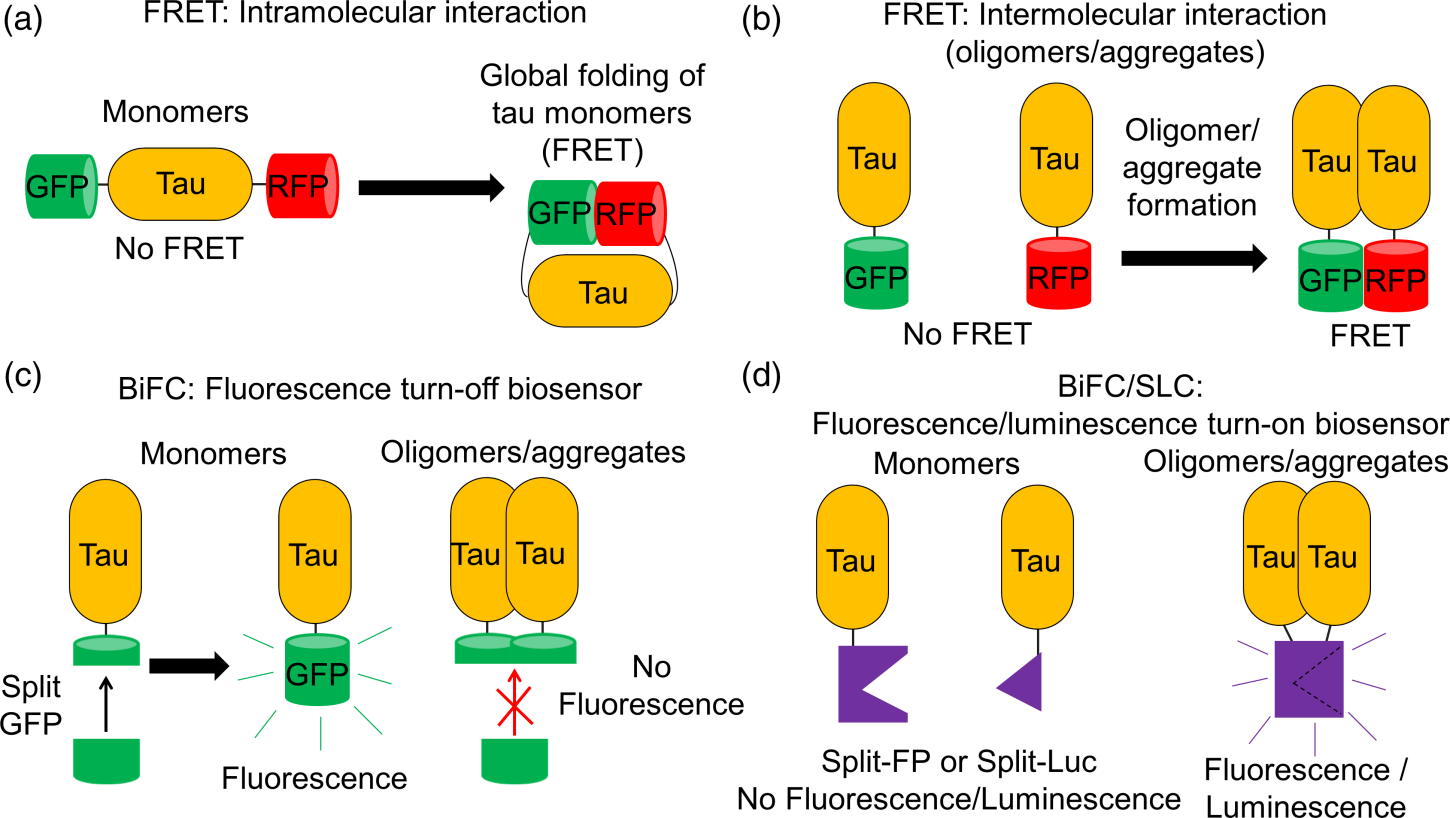
Schematic representation of tau biosensors based on fluorescence resonance energy transfer (FRET), bimolecular fluorescence complementation (BiFC), or split luciferase complementation (SLC) for monitoring intramolecular and intermolecular tau interactions in living cells. (a) Tau intramolecular FRET biosensor where FRET is observed when there is global folding of wild‐type (WT) monomeric tau.^58^ (b) Tau intermolecular FRET biosensors where FRET is observed when tau oligomers or aggregates are formed. WT tau is used for the formation of non‐β‐sheet soluble tau oligomers and tau repeat domain (tauRD) with P301S mutation or truncated tau is used for the formation of β‐sheet insoluble tau aggregates.^55^, ^58^, ^66^ (c) BiFC tau fluorescence turn‐off biosensor where fluorescence is absent when there is tau oligomerization or aggregation.^78^, ^79^ (d) BiFC/SLC tau fluorescence/luminescence turn‐on biosensor where fluorescence or luminescence is present when tau oligomers or aggregates are formed.^80^, ^87^, ^89^ Tau oligomer is drawn as a dimer for illustration but it can be any species more than a dimer (≥2‐mers)

Several cell‐based studies have illustrated that tau oligomerization and accumulation in cells result in neurotoxicity and cell death.^25^, ^47^, ^48^, ^49^ Cellular assays are also responsive to indirect pathways and various posttranslational modifications such as phosphorylation and methylation,^6^ which play key roles in determining the formation of toxic tau oligomers.^50^ Therefore, the biophysical and biochemical characterization of soluble tau oligomeric species in the cellular context is necessary and will provide insights to the disease mechanism.^51^, ^52^ However, these approaches are far less adopted, in part because of the lack of tools available for such studies. In this review, we will present the use of different biophysical strategies such as fluorescence resonance energy transfer (FRET), bimolecular fluorescence complementation (BiFC), and split luciferase complementation (SLC), to investigate the spontaneous formation of tau oligomers or inducers‐stimulated tau oligomerization and aggregation in living cells (Figure 2). These strategies allow us to achieve a spatiotemporal resolution of intramolecular interactions present in the ensembles of tau oligomers and a range of intermolecular tau–tau interactions, from soluble dimers and oligomers to large aggregates. A list of cellular tau biosensors engineered based on different strategies and their respective abilities to detect the formation of soluble oligomers and/or insoluble aggregates as well as seeding are summarized (Table 1). We will also discuss the applications of these cellular biosensors to study cell‐to‐cell propagation of tau pathology and their potential as translational tools for biomarker development and therapeutic discovery. This information will provide insights to the understanding of the heterogeneity of tau oligomers and their role as molecular targets for therapeutic development of AD and related tauopathies.

**TABLE 1:** Cellular biosensor technologies to study tau oligomerization and aggregation

Methods	Tau isoforms	Mutants	Tags	Models	Inducers	Aggregate types	Detect seeding	Reference
FRET	0N4R	WT	CFP/YFP	HEK293	Spontaneous formation	Soluble oligomers	—	55
	0N4R (Δ421‐441)				GSK3β	Insoluble aggregates		
	2N4R	WT	GFP/RFP	HEK293	Spontaneous formation	Soluble oligomers	Yes	58
		P301L		SH‐SY5Y	Forskolin			
	K18 (RD)	WT ΔK280	CFP/YFP	HEK293	WT K18 PFFs	Insoluble aggregates	Yes	66 67
		P301L/V337M						
		P301S						
		ΔK280/I227P/I308P						
BiFC	0N4R 0N4R (Δ421‐441) K18 (RD)	WT ΔK280 ΔK280/I227P/I308P	Split GFP	HEK293	Spontaneous formation GSK3β	Soluble oligomers Insoluble aggregates	—	78 79
	2N4R	WT	Split Venus	HEK293	Spontaneous formation	Soluble oligomers	Yes	80 84 99 111

				SH‐SY5Y	Forskolin	Insoluble aggregates		
					Okadaic acid			
					K18‐P301L			
	2N4R	P301L	Split Venus	Mouse model	Spontaneous formation	Soluble oligomers Insoluble aggregates	Yes	87
SLC	2N4R	WT	Split gLuc	HEK293	Spontaneous formation	Soluble oligomers	Yes	89

					Heparin			
					WT PFFs			
					Mouse brain lysates (tauP301L aggregates)			
	2N4R	WT	Split NanoLuc	HuH‐7	Spontaneous formation	Soluble oligomers	—	92
	K18 (RD)	P301S	Split cbgLuc	HEK293	Spontaneous formation	Soluble oligomers	Yes	94 95
		ΔK280		N2a	WT K18 oligomers WT K18 PFFs	Insoluble aggregates		

Abbreviations: BiFC, bimolecular fluorescence complementation; FRET, fluorescence resonance energy transfer; GSK3β, glycogen synthase kinase 3 beta; HEK293, human embryonic kidney 293; PFFs, preformed fibrils; SLC, split luciferase complementation; WT, wild‐type.

## 2 FRET‐BASED BIOSENSORS

FRET is a process by which energy is transferred from a donor fluorophore to an acceptor fluorophore when an FRET pair, such as green and red (GFP/RFP) or cyan and yellow (CFP/YFP) fluorescent proteins, is in close proximity. An FRET pair is individually fused to interacting proteins and exhibits FRET when they are brought to a distance of less than 10 nm apart.^53^, ^54^ The different forms of cellular tau FRET biosensors engineered (Table 1) and the different methods of FRET measurements will be discussed below.

### 2.1 Acceptor photobleaching‐based FRET microscopy

**FIGURE 3:**
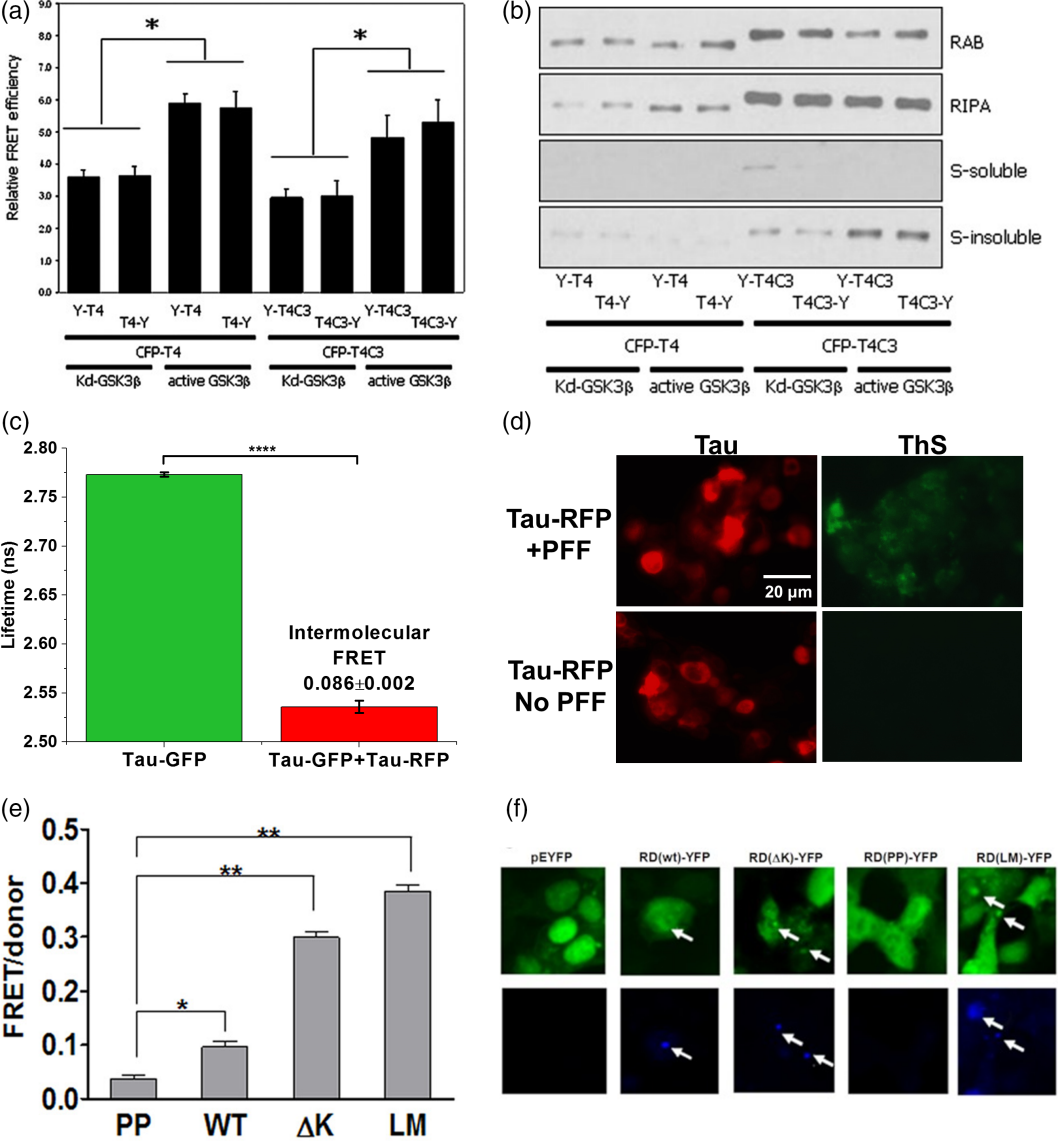
Cellular tau fluorescence resonance energy transfer (FRET) biosensors to examine tau oligomerization and aggregation. (a) Through acceptor photobleaching method, basal FRET is observed in both 0N4R wild‐type (WT) tau (T4) and Δ421‐441 truncated tau (T4C3) CFP/YFP FRET biosensors in the presence of kinase‐dead (Kd) glycogen synthase kinase 3 beta (GSK3β) (absence of GSK3β kinase activity). FRET efficiencies are increased with treatment of active GSK3β.^55^ (b) T4 FRET biosensors form soluble tau species or oligomers while T4C3 FRET biosensors form insoluble aggregates in the presence of active GSK3β.^55^ (c) Lifetime measurement of the WT 2N4R tau intermolecular GFP/RFP FRET biosensor shows a shorter lifetime in tau‐GFP/RFP (donor‐acceptor) sample as compared to tau‐GFP (donor) sample which exhibits efficient FRET.^58^ (d) Thioflavin‐S (ThS) staining of human embryonic kidney 293 (HEK293) cells expressing tau‐RFP (same total DNA concentration as the FRET biosensor) shows a positive signal with treatment of tau preformed fibrils (PFF) as a positive control. In the absence of PFF, there is no ThS signal, indicating formation of nonβ‐sheet soluble oligomers by the WT 2N4R tau FRET biosensor.^58^ (e) FRET measurement of tauRD CFP/YFP FRET biosensors with different tau variants expressed in HEK293 cells. PP refers to ΔK280/I227P/I308P mutations, ΔK refers to ΔK280, and LM refers to P301L/V337M mutations.^66^ (f) Formation of inclusions are observed in WT, ΔK and LM variants of the tauRD CFP/YFP FRET biosensors, as characterized by positive staining of X‐34 which is an amyloid‐specific dye.^66^ Permissions obtained from References 55, 58, and 66

The FRET technique to investigate tau–tau intermolecular interaction was initially introduced by expressing CFP or YFP tagged WT 0N4R or caspase‐cleaved (ΔAA421‐441) tau proteins at either N‐ or C‐terminal in living human embryonic kidney 293 (HEK293) cells.^55^ Through the acceptor photobleaching method, similar levels of FRET signals are detected in both WT CFP‐tau/YFP‐tau and CFP‐tau/tau‐YFP expressing cells in the absence of aggregation inducers (Figure 3(a)). This indicates spontaneous oligomerization of WT tau, independent of the positions of fluorophore tagging, as an initial step in the aggregation cascade. The similar FRET observed from differential fluorophore tagging suggests that tau is adopting different conformations in the formation of oligomers and the presence of conformational ensembles in tau oligomerization. In the same study, a higher FRET signal is detected in the presence of glycogen synthase kinase 3 beta (GSK3β) co‐expression with WT tau, illustrating a higher aggregation propensity of hyperphosphorylated tau which is confirmed by biochemical assays. Interestingly, a similar level of higher FRET is observed in caspase‐cleaved tau which is more resistant to GSK3β‐mediated phosphorylation but known to form insoluble thioflavin‐S (ThS) positive inclusions in cells (Figure 3(a)).^56^ Indeed, the analysis of cell lysates shows that WT tau forms sarkosyl soluble fractions in native cellular environment, while the caspase‐cleaved tau proteins are sarkosyl insoluble (Figure 3(b)), indicating that FRET originates from respective tau species. This is consistent with previous studies showing that truncated tau aggregates more rapidly than WT tau and has been reported to promote fibril formation, especially under aberrant phosphorylation.^57^ It is worth emphasizing that the similar FRET levels obtained with different species and treatment conditions suggests the need to stringently resolve the exact species that corresponds to the observed FRET in order to accurately interpret the signals.

### 2.2 Lifetime‐based FRET measurement

Recently, we engineered two cellular WT 2N4R tau FRET biosensors to monitor intramolecular (GFP‐tau‐RFP) (Figure 2(a)) and intermolecular (tau‐GFP/tau‐RFP) (Figure 2(b)) interactions. We show efficient FRET signals through lifetime measurements in HEK293 cells expressing the biosensors,^58^ recapitulating the global folding of the tau proteins in the intramolecular system^59^, ^60^ and their spontaneous oligomerization in the intermolecular system (Figure 3(c)). We note that the FRET efficiency reflects the average of the ensemble intermolecular and intramolecular proximity between tau molecules which potentially contains multiple different conformations and interaction states. More importantly, there is no puncta formation in cells expressing the WT tau biosensors and they are not ThS positive, hence confirming that these are soluble tau oligomers and do not contain β‐sheet species (Figure 3(d)).^58^ This is also consistent with previous studies showing that WT tau is resistant to fibrillization and forms mainly tau oligomers.^29^ Furthermore, we also expressed fluorophore‐tagged tau with P301L mutation (tauP301L‐GFP/tauP301L‐RFP) in both HEK293 and SH‐SY5Y cells and show that higher FRET signal is observed in tauP301L FRET biosensor than the WT tau biosensor, indicating the higher propensity of oligomerization for tauP301L.^58^ Importantly, we have also observed an increase in FRET with treatment of recombinant tau proteins to the biosensor cells, suggesting the possibility of using our biosensors in seeding experiments. As overexpression of fluorophore‐tagged tau in HEK293 cells does not illustrate toxicity, we overexpressed unlabeled WT tau or tauP301L in SH‐SY5Y cells to study the effect of tau oligomers in cell cytotoxicity.^58^ Overexpression of WT tau does not induce significant cell cytotoxicity but overexpression of tauP301L in SH‐SY5Y cells induces significant toxicity, consistent with other observations.^61^, ^62^, ^63^, ^64^ Interestingly, it has been shown that overexpression of tauP301L does not induce fibril formation in SH‐SY5Y cells,^65^ suggesting that tauP301L‐induced toxicity is due to toxic oligomers.

### 2.3 Flow cytometry‐based FRET measurement

With a primary goal of detecting uptake, seeding and propagation of toxic tau species, the Diamond group developed a groundbreaking tau FRET biosensor made up of tau repeat domains (RD) (tauRD‐CFP/YFP or tauRD FRET biosensor). Different variants and mutations of tau, including WT, ∆K280 (∆K), P301L/V337M (LM), ∆K280/I227P/I308P (PP) show varying FRET signals (Figure 3(e)).^66^ Importantly, distinct puncta and evident thioflavin positive fibrillar species are present, indicating the formation of β‐sheet aggregates by tauRD FRET biosensor (Figure 3(f)), but no toxicity is observed. The unlabeled tauRD aggregates are also shown to be released from one cell population and taken up by recipient tau biosensor cells to induce aggregation as illustrated through an increase in FRET. This indicates seeding^67^, ^68^ and the propagation of seeds between cells.^66^, ^69^ The use of the tauRD FRET biosensor has also been extended to the study of the initiation of pathological aggregation beginning with conversion of inert tau monomer to a seed‐competent form based on increasing accessibility of the hexapeptide motifs (VQIINK/VQIVYK) that promote aggregation.^21^ This is consistent to a report that pathogenic tau mutations, alternative splicing and proline isomerization are all capable of destabilizing the local structure proximal to the hexapeptide motifs and triggering spontaneous aggregation as illustrated by an increase in FRET of the tauRD biosensor.^70^ More recently, the tauRD FRET biosensor is applied to study the seeding capability of tau oligomers. Purified oligomeric assemblies containing 3‐mer, ∼10‐mer, and ∼20‐mer as well as fibrils have been shown to increase FRET of the tauRD biosensor.^71^ Treatment of heparin or a heparinoid compound (SN7‐13) inhibits seeding by both oligomeric and fibrillar species and reduces FRET of the biosensor.^71^

The development of these cellular FRET biosensor technologies provide the platforms to study the aggregation cascade of tau proteins, including monomers (intramolecular doubly labeled tau or single‐molecule FRET^72^), oligomers (intermolecular WT tau biosensor) and fibrillar species (intermolecular tauRD biosensor). Some of these systems, especially the intramolecular biosensors, have also been used to study tau‐microtubule interactions^73^, ^74^, ^75^ as well as the detachment of tau from microtubule and the subsequent formation of oligomers and aggregates.^76^

## 3 SPLIT FLUORESCENT PROTEIN COMPLEMENTATION OR BiFC‐BASED BIOSENSORS

An important consideration in selecting a fluorescence‐based technique to quantify PPIs is to ensure that the use of fluorescent proteins does not obstruct the biological interactions between the proteins of interest. While FRET is one of the prominent approaches to investigate protein interactions, it requires large fluorescent protein fusion which may cause steric hindrance that potentially results in inaccurate measurement of the key interactions. To minimize the size of fluorescent protein tagging, the split fluorescent protein technique or BiFC is introduced (Table 1) where a fluorescent protein is split into two nonfluorescent fragments to reduce the size of fusion as well as background fluorescence.^77^ Two types of BiFC‐based biosensors are available: (1) BiFC fluorescence turn‐off biosensor where tau monomers exhibit fluorescence and aggregation abolishes the fluorescence (Figure 2(c)), and (2) BiFC fluorescence turn‐on biosensor where the nonfluorescent constituents are tagged to tau proteins and fluorescence is observed when tau self‐associates (Figure 2(d)).

### 3.1 Split‐GFP complementation (fluorescence turn‐off biosensor)

To create a tau fluorescence turn‐off biosensor, Johnson group adopted a split‐GFP complementation approach by fusing WT 0N4R tau protein directly to a small nonfluorescent GFP fragment (GFP11), and co‐expressing in HEK cells with a large nonfluorescent GFP fragment (GFP1–10).^78^, ^79^ When tau exists as a monomer or low degree aggregate, the complementary large GFP fragment is able to access the small GFP fragment fused to tau, assembling the fluorescently active GFP. The reconstitution of active GFP is prohibited primarily due to steric hindrance when tau aggregates, leading to a decrease in GFP fluorescence intensity in cells.^78^, ^79^ The complementation of the tau‐GFP occurs in a concentration‐dependent and linear manner, illustrating the sensitivity of this assay to monitor the fusion of nonfluorescent GFP and hence quantifying tau oligomerization and aggregation. Different tau fluorescence turn‐off biosensors have been generated based on various tau constructs, including K18‐WT, K18‐ΔK, and K18‐ΔK‐Proline‐Mutant. The basal fluorescence for all constructs increase to a maximum after 48 h, with K18‐ΔK being the dimmest, K18‐ΔK‐Proline‐Mutant being the brightest and K18‐WT with intermediate intensity level. This confirms the pro‐aggregation effect of K18‐ΔK and aggregation resistance of K18‐ΔK‐Proline‐Mutant as compared to K18‐WT.^78^ This result indicates that there is spontaneous formation of tau oligomers in the basal level after transfection. There is also a general decrease in the fluorescent intensity across all constructs with time, which indicates that more oligomerization and aggregation occurs with longer transfection period.^78^

Besides WT tau, the fluorescence turn‐off approach was also applied to truncated tau (ΔAA421‐441) and tau‐2EC with two Ser‐to‐Glu mutations at Ser396/S404 to mimic phosphorylation of these sites. The results show that truncated tau and tau‐2EC proteins are accumulating in cells to a greater extent with reduced GFP fluorescence as compared to WT tau in the absence of phosphorylation inducers.^78^ The presence of active GSK3β significantly increases the GFP intensity of WT, which strikingly corresponds directly to the increased expression of WT tau as illustrated through immunoblots. An increase in the fluorescence intensity of the turn‐off biosensor should correspond to less aggregation with more monomers, but this is complicated with an increase in tau expression. Therefore, although active GSK3β is used, it is unclear from the fluorescence turn‐off biosensor whether extensive phosphorylation of WT tau leads to an enhanced oligomerization or aggregation as these should have led to a decrease in GFP intensity. Conversely, no change in GFP intensity is observed for truncated tau regardless of phosphorylation and no change in tau expression is observed.^78^ This is a conflicting observation from the FRET study where the truncated tau shows higher FRET signal with active GSK3β expression, which we will expect a decrease in GFP intensity in the split‐GFP tau biosensor to indicate increasing aggregation; however, this is not observed in this study. Interestingly, tau‐2EC, which is pseudophosphorylated at S396/S404 and is not efficiently further phosphorylated by active GSK3β, exhibited significantly decreased GFP intensity in the presence of active GSK3β, indicating that there is increased aggregation.^78^ This is suggested to be due to the formation of sarkosyl insoluble aggregates and enhanced phosphorylation at other residues besides S396/S404. We should also note that this method is only feasible when there is an initial self‐assembly between the two parts of the split‐GFP to form monomer signals of GFP fluorescence, but the intrinsic affinity between these two GFP parts may affect the aggregation propensity of the protein of interest.

### 3.2 Split‐Venus BiFC (fluorescence turn‐on biosensor)

In a BiFC fluorescence turn‐on biosensor (hereinafter refers to as a tau‐BiFC biosensor), fluorescence is exhibited when there are interactions between the protein of interest. This can overcome the intrinsic limitations to monitor the more transient early‐stage spontaneous formation of soluble tau intermediates in the fluorescence turn‐off biosensor. An eminent example of the tau‐BiFC biosensor is the split‐Venus‐based BiFC system established by the Kim group where the two nonfluorescent N‐ and C‐terminal fragments of the Venus fluorescent protein are individually fused to tau, and tau self‐assembly turns on the Venus fluorescence.^80^ The Venus protein is a variant of YFP and is well suited for achieving spatiotemporal resolution of tau assembly because (1) it has fast and efficient maturation, (2) its self‐assembly rate is low compared to that of other BiFC pairs, and (3) the fluorescence intensity of Venus‐based BiFC is higher than that of EYFP‐based BiFC.^81^

**FIGURE 4:**
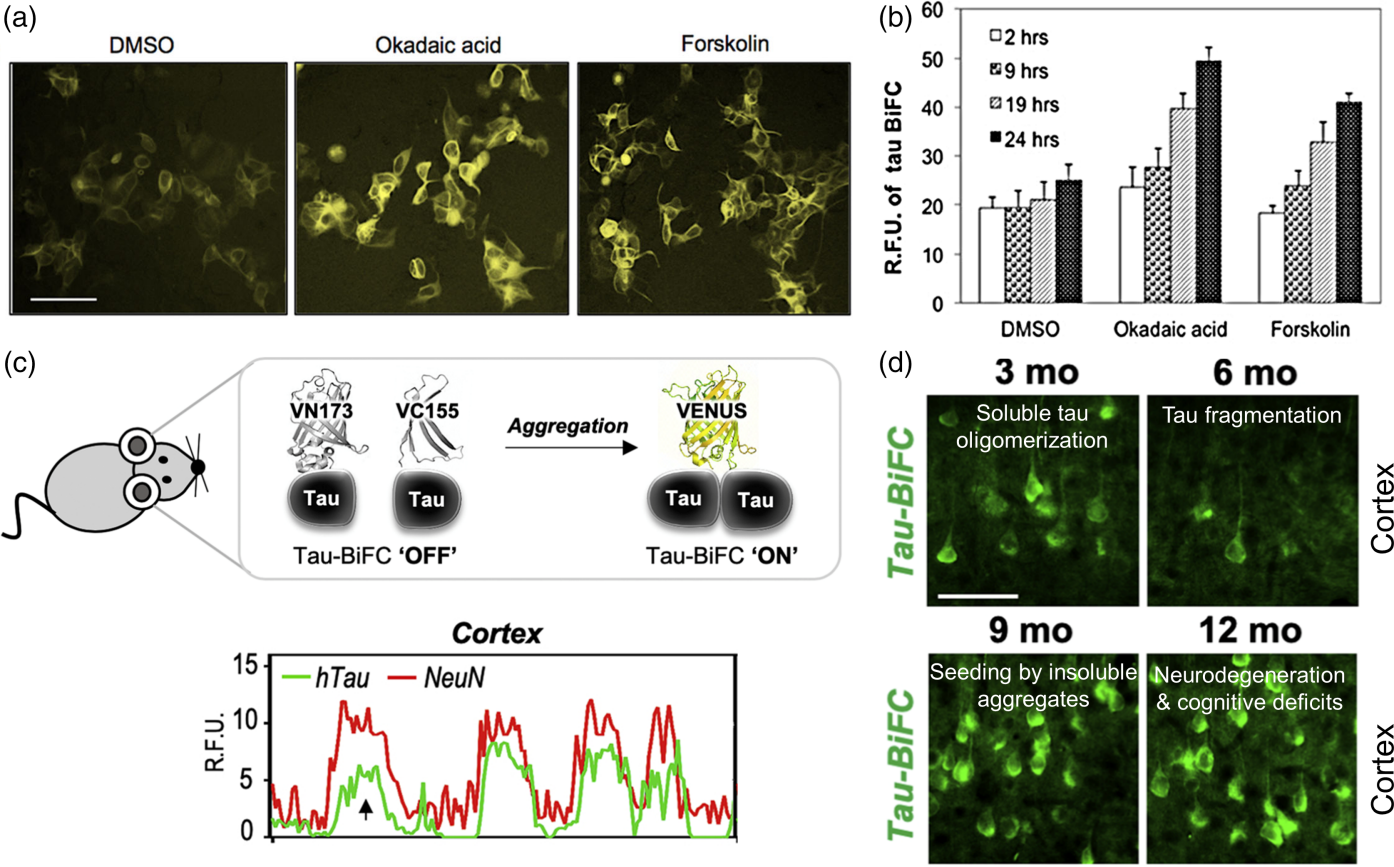
Bimolecular fluorescence complementation (BiFC) biosensors to evaluate tau oligomerization and aggregation in cells and in a transgenic mouse model. (a) Fluorescence microscopy images of Venus‐based tau‐BiFC fluorescence turn‐on biosensor illustrating increased fluorescence complementation with treatment of phosphorylation inducers (okadiac acid at 30 nM and forskolin at 20 μM, scale bar = 200 μm).^80^ (b) Quantification of Venus‐based tau‐BiFC biosensor shows an increasing fluorescence signals with longer incubation time in cells treated both with and without phosphorylation inducers.^80^ (c) Schematic of human tauP301L‐BiFC transgenic mouse model which exhibits Venus fluorescence upon tau oligomerization and aggregation. Correlation plot of the immunofluorescence intensity profile between tau (green) and NeuN (red) shows the expression of tau in the cortex.^87^ (d) Representative images of fluorescence complementation in tauP301L‐BiFC mouse cortical regions at ages 3–12 months corresponding to different tau pathologies (scale bar = 50 μm).^87^ Permissions obtained from References 80 and 87

The Venus‐based tau‐BiFC biosensor is generated by fusing WT 2N4R human tau to the N‐ (1–172 amino acids, VN173) or the C‐terminal (155–238 amino acids, VC155) fragment of Venus fluorescent protein, followed by stable expression of these DNA constructs in HEK293 cells, with tau‐GFP expressing cells used as a control and comparison.^80^ Interestingly, a basal fluorescence intensity about 20% of that of the tau‐GFP expressing cells is observed in the tau‐BiFC cells on the basis of similar level of protein expression. This indicates that there are some spontaneous tau dimeric or oligomeric interactions under physiological conditions, with majority of the tau molecules exist as monomers.^80^ Treatment of small molecules okadaic acid (30 nM) or forskolin (20 μM) induces tau hyperphosphorylation and increases in tau–tau interactions. This is illustrated by an enhanced fluorescence intensity of the biosensor cells (Figure 4(a)) which follows a time‐course‐dependent manner (Figure 4(b)).^80^ Interestingly, in both basal and hyperphosphorylation conditions, despite changes in the fluorescence levels, there are no distinct changes in the cell morphology and no presence of puncta which corresponds to higher molecular weight aggregates, except some neurite‐like structures. This suggests that soluble tau dimers and oligomers are present in the biosensor cells under both conditions. Cells expressing the Venus‐based tau‐BiFC biosensor have been applied to study the regulation of tau oligomerization by different mechanisms, which will be discussed below.

Several studies have suggested that 26S proteasome activity may be beneficial to neuronal cells as they play a key role in degrading abnormally accumulated protein aggregates that are implicated in neurodegeneration.^82^ To be delivered into cells, purified proteasomes have been loaded onto novel mesoporous silica nanoparticles, which act as a proteasome transporte*r*. Tau oligomerization is induced by okadaic acid, which shows a high fluorescence complementation in tau‐BiFC biosensor cells. Treatment with proteasome–MSNPN complexes illustrates less tau aggregation with a significantly lower fluorescence complementation compared with cells treated with MSNPN alone which do not show significant change in fluorescence complementation.^83^ Therefore, cells treated with exogenous proteasomes are more efficient in degrading overexpressed human tau than endogenous proteasomal substrates. This results in a decrease in tau oligomers and aggregates, and a reduced proteotoxic stress caused by tau and reactive oxygen species. Cells expressing tau‐BiFC biosensor are also used to study tau aggregation in ATP dyshomeostasis.^84^ To examine whether an increased level of ATP affects the extent of tau self‐assembly, the tau‐BiFC biosensor cells are treated with TNFα/CHX to induce an increase in the intracellular ATP levels. A significant increase in the average fluorescence intensity of tau‐BiFC by 60–70% in both HEK293 and SH‐SY5Y cells indicates an enhanced intracellular tau aggregation with upregulation of ATP.^84^

Recent studies have also shown that O‐linked β‐*N*‐acetylglucosamine (O‐GlcNAc) reduces tau hyperphosphorylation by protecting the tau phosphorylation sites in normal brain. In pathological condition, tau is de‐glycosylated and becomes a substrate for kinases.^85^ Two enzymes, O‐GlcNAc transferase (OGT) and O‐GlcNAcase (OGA), are responsible for the catalysis of the addition and removal of O‐GlcNAc, respectively. As the tau‐BiFC biosensor cells are capable of revealing the aggregation potential of tau based on its phosphorylation states, they have been used to study the role of OGA and OGT on tau aggregation. Inhibition of OGA by Thiamet G decreases aggregation and leads to a 30% reduction in the complementation response. On the other hand, inhibition of OGT by BZX2 increases aggregation and results in a nearly twofold elevation in biosensor fluorescence.^86^ These results confirm the counter‐regulatory mechanism of OGA and OGT in tau pathology.

To bring this important BiFC tool in vivo to directly monitor tau self‐assembly in the mouse brain, a novel tau transgenic mouse expressing tauP301L‐BiFC fluorescence turn‐on biosensor was recently generated (Figure 4(c)).^87^ Initially, there is a significant increase in BiFC fluorescence in the mouse brain in the first 3 months, corresponding to the initial spontaneous formation of tau oligomers. At this time, most neurons in tauP301L‐BiFC mice are also shown to express tau in the cortex (Figure 4(c)). At 6 months, tau cleavage occurs which results in a dramatic decrease in BiFC fluorescence. The truncated tau species then serves as a seed for triggering further insoluble tau aggregation, resulting in a subsequent increase in BiFC fluorescence from 9 months. Above 12 months of age, neurodegeneration and cognitive dysfunctions are observed in the tauP301L‐BiFC mice (Figure 4(d)).^87^ This in vivo full‐length tauP301L‐BiFC fluorescence turn‐on mouse model recapitulates in vitro observation of the early stages of tau pathology such as oligomerization, truncation, nucleation and fibrillization.^55^, ^57^, ^58^, ^66^, ^80^

## 4 SLC‐BASED BIOSENSORS

To improve on the BiFC assay, tau SLC assays are developed. SLC assays possess enormous dynamic range with superior detection sensitivity and more dynamic reversibility of the complementation.^88^ Examples of SLC assays are detailed below with different types of split luciferase used in each assay (Table 1).

### 4.1 Gaussia‐Luc SLC

**FIGURE 5:**
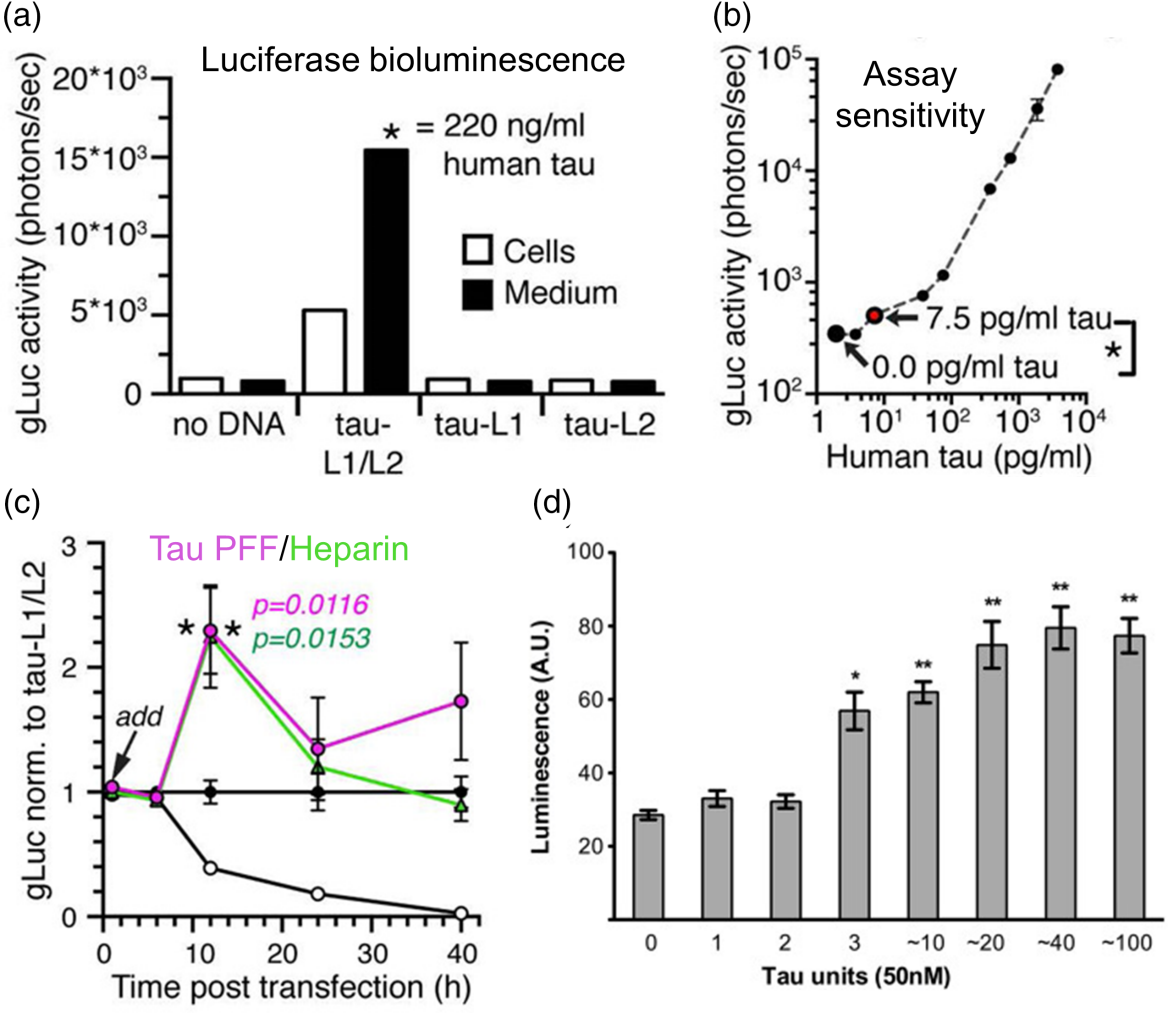
Characterization of split luciferase complementation (SLC) biosensors and their ability to measure the minimum tau units to induce aggregation. (a) Split Gaussia luciferase (split‐gLuc) complementation biosensor illustrating increased complementation of luciferase bioluminescence both in human embryonic kidney 293 (HEK293) cells expressing the split gLuc plasmids (tau‐L1/L2) and in the culture medium where tau oligomers are released by the transfected cells.^89^ (d) Linear correlation between split‐gLuc activity and tau concentration indicating an assay sensitivity of 7.5 pg/ml tau‐L1/L2 equivalent to 0.16 nM full‐length tau as characterized by human total tau ELISA.^89^ (c) Treatment of tau preformed fibrils (PFF) and heparin to split‐gLuc biosensor accelerates oligomer formation in cell culture medium after 12 h with a subsequent decrease in the luciferase activity after 24 h.^89^ (d) Treatment of tauRD oligomers with a minimum of 3 units to HEK293 cells expressing RD‐Nluc/Cluc increases click beetle green luciferase signals.^94^ Permissions obtained from References 89 and 94

To generate an SLC assay, the Hyman group used WT 2N4R tau to fuse to either the N‐ or the C‐terminal region of Gaussia luciferase (gLuc), forming a split‐gLuc.^89^ The formation of tau oligomers enables the complementation of two separate parts of split‐gLuc, namely tau‐L1 (residues 1–92) and tau‐L2 (residues 93–163), which then reconstitutes gLuc activity. Robust gLuc activity is observed in HEK293 cells with co‐expression of tau‐L1 and tau‐L2, as well as in the conditioned medium after 40 h of transfection (Figure 5(a)), without observed toxicity from the tau split‐gLuc expression.^89^ The control, L1 and L2 only without fusing to tau, does not result in luciferase activity, indicating specific tau–tau self‐association and oligomerization in the SLC assay. Linear correlation between split‐gLuc activity and tau concentration in conditioned medium indicates an excellent assay sensitivity of 7.5 pg/ml tau‐L1/L2 which is equivalent to 0.16 nM full‐length tau as characterized by human total tau ELISA (Figure 5(b)). More importantly, these tau species are not thioflavin‐T (ThT) positive, indicating that tau‐L1/L2 activity originates mostly from dimers and oligomers but not insoluble tau fibrillar species with β‐sheet.^89^ Furthermore, the tau‐L1/L2 complementation is enhanced with addition of aggregation inducers, including heparin, preformed fibrils,^90^ and mouse brain lysates,^91^ to tau‐L1/L2 transfected HEK293 cells (Figure 5(c)). The gLuc activity reaches a maximum after 12–24 h of inducer treatment and subsequently decreases to the nontreated basal level after 48 h.^89^ This is an interesting observation as these inducers will typically result in the formation of irreversible and insoluble β‐sheet oligomers and aggregates,^4^ which should not result in a decrease in luciferase signals. The authors suggest that this could be due to accelerated oligomerization kinetics upon different aggregation inductions although the overall oligomeric contents remain the same. We also speculate that this temporary spike in luciferase complementation is probably due to spontaneously formed reversible, soluble and off‐pathway tau oligomers as they do not proceed to form β‐sheet aggregates, although the extent in which the inducers trigger cellular tau oligomerization remains to be investigated. It is also reported that the stable intracellular tau oligomers can be released and internalized by split‐gLuc biosensor cells, constituting the cell‐to‐cell spreading phenomena in tau pathology and resulting in increased luciferase bioluminescence signals.^89^

### 4.2 NanoLuc SLC

Using a similar approach, a NanoLuc SLC assay is generated with N‐ and C‐terminal of WT 2N4R tau fused to two different NanoLuc luciferase subunits (an 18 kDa polypeptide (large Bit) and a 1.3 kDa peptide (small Bit)) respectively.^92^ NanoLuc activity is observed in human liver HuH‐7 cells transfected with the two tau‐NanoLuc fusion constructs for 24 h, illustrating the reconstitution of luciferase activity based on tau protein self‐association and oligomerization.^92^ The NanoLuc activity is reduced with treatment of protein kinase inhibitors, suggesting that the tau oligomerization is initiated by self‐association of phosphorylated tau monomers and that the kinase inhibitors are either directly inhibiting tau phosphorylation or indirectly affecting alternative mechanisms.^92^

### 4.3 Click beetle green‐Luc SLC

In another study to investigate tau propagation and spontaneous internalization for cell‐to‐cell spreading of tau pathology, a tau SLC assay based on click beetle green luciferase (cbgLuc) is developed.^93^ The N‐ and C‐terminal halves of the cbgLuc are fused to the C‐terminal of the tauRD containing P301S mutation, and the tau split‐cbgLuc illustrates a basal cbgLuc activity upon spontaneous tau dimerization and oligomerization.^94^, ^95^ Interestingly, the luciferase signal is increased with treatment of exogenous tau oligomers (*n* ≥ 3) and fibrils, suggesting the minimal tau assembly capable of spontaneous cell uptake and seeding (Figure 5(d)).^94^

## 5 TRANSLATIONAL APPLICATIONS OF CELL‐BASED TAU BIOSENSORS

The cellular tau biosensors are continuously being used in biomarker development as well as therapeutic discovery such as identification of small molecules or antibodies that disrupt toxic tau interactions or alter tau conformational ensembles. In this section, we will discuss the sensitivity of each type of tau biosensor in detecting seeding activity and examples of tau biosensors used as translational tools in high‐throughput screening (HTS) drug discovery.

### 5.1 Detection of seeding activity as a biomarker for AD pathology

High seeding activity in a biological sample can be a new biomarker for a subset of subjects that are more likely to develop symptoms of pathological tau aggregation. The cellular tau biosensors have been widely used to characterize the seeding capability of in vitro protein samples and biological samples from both mice and human.

#### 5.1.1 TauRD FRET biosensor

The tauRD FRET biosensor is highly sensitive and specific to the detection of isolated tau oligomeric species and fibrillar aggregates from human patients and AD transgenic mouse brains that are seed‐competent.^96^, ^97^, ^98^ Specifically, tauRD FRET biosensor has been used to test the tau seeding capacity of the soluble high‐molecular‐weight brain fraction containing mostly seed‐competent oligomeric species from AD patients.^97^, ^98^ The FRET assay readily discriminates diseased and aged control brains,^97^ and further illustrates heterogeneous seeding properties across the patients as characterized by different extents of FRET increase.^98^ In another study, brain‐derived tau oligomers or oligomeric tau seeds from AD, dementia with Lewy bodies, and progressive supranuclear palsy patients have been shown to significantly increase FRET of tauRD biosensor with clear formation of tau inclusion as observed by immunofluorescence microscopy.^18^

#### 5.1.2 Tau‐BiFC biosensor

The seeding capacity of the tau‐BiFC biosensor was evaluated with treatment of the preaggregates and aggregates of purified proteins of K18‐WT and K18‐P301L to the cell culture medium of tau‐BiFC biosensor cells.^99^ The preaggregates and aggregates of K18‐P301L increase the fluorescence intensity of cells by twofold while K18‐WT does not induce any increase in BiFC fluorescence under any aggregation conditions.^99^ This suggests the greater propagation and internalization of the K18‐P301L than the K18‐WT.

#### 5.1.3 TauRDΔK SLC biosensor

To examine whether tau containing exosomes derived from the cerebrospinal fluid (CSF) of AD patients are seed‐competent, tauRDΔK SLC biosensor was generated with two halves of luciferase fusing to tauRD containing ΔK280 mutation and expressed in N2a cells.^28^ Tau containing exosomes dramatically increase the luciferase bioluminescence signal by about 50% for AD patients and 40% for healthy controls, corresponding to an increase in tauRDΔK aggregation.^28^ While exosomes from AD patients induced slightly higher aggregation than the controls, it is important to note that the difference is not statistically significant.^28^ This suggests that exosomes from both AD and control CSF may contain different species of tau oligomers which are capable of triggering tau aggregation to a different extent, recapitulating the heterogeneity in tau oligomers.^98^

**FIGURE 6:**
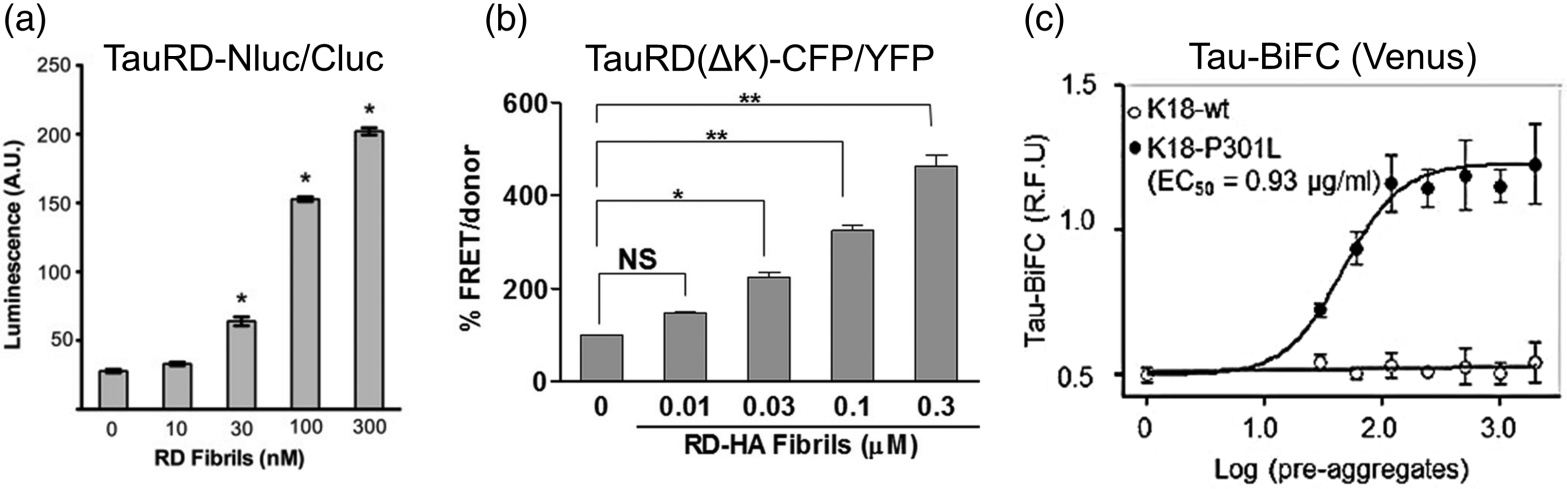
Comparison of the sensitivity of the different cellular biosensors in detecting tau seeded aggregation. (a) Treatment of an increasing concentration of tauRD fibrils to human embryonic kidney 293 (HEK293) cells expressing tauRD‐Nluc/Cluc induces aggregation of the split luciferase complementation (SLC) biosensor as shown by an increase in the luciferase luminescence.^94^ (b) Treatment of an increasing concentration of tauRD fibrils to HEK293 cells expressing tauRD(ΔK)‐CFP/YFP induces aggregation of the fluorescence resonance energy transfer (FRET) biosensor. The ΔK indicates ΔK280 variant of tau.^66^ (c) Dose‐dependent increase in tau‐bimolecular fluorescence complementation (BiFC) fluorescence induced with tau K18‐P301L proteins in Venus‐based tau‐BiFC fluorescence turn‐on biosensor expressed in HEK293 cells.^99^ Permissions obtained from References 94, 66, and 99

By comparing the efficacy of these three types of biosensors in detecting seeding activity, tau seeds activate all of them with a half‐maximal effective concentration (EC_50_) between 0.03 and 0.1 μM, although they have different dynamic ranges of detection (Figure 6). TauRDΔK SLC biosensor has the greatest dynamic range with an eightfold increase in signal between nontreated and treatment of the highest concentration of tauRD seeds (Figure 6(a)).^94^ This is followed by a fourfold signal increase in tauRD FRET biosensor with treatment of tauRD seeds (Figure 6(b)^66^ and a twofold signal increase in tau‐BiFC biosensor with treatment of K18‐P301L tau (Figure 6(c)).^99^ However, we should note that the seeds used in these studies are different, which might contribute to the difference in the sensitivity or fold change observed.

### 5.2 HTS drug discovery

Fluorescence and bioluminescence biosensors constitute attractive and powerful tools for drug discovery campaigns, from HTS assays, to optimization of lead compounds, and preclinical evaluation of candidate drugs. In the context of tau oligomerization and aggregation, tau biosensors have been used in HTS assays to identify small molecules that interfere with tau–tau interactions, conformational changes of tau and other biological processes that alter the propensity of tau aggregation such as tau phosphorylation.

#### 5.2.1 WT 2N4R tau FRET biosensor

**FIGURE 7:**
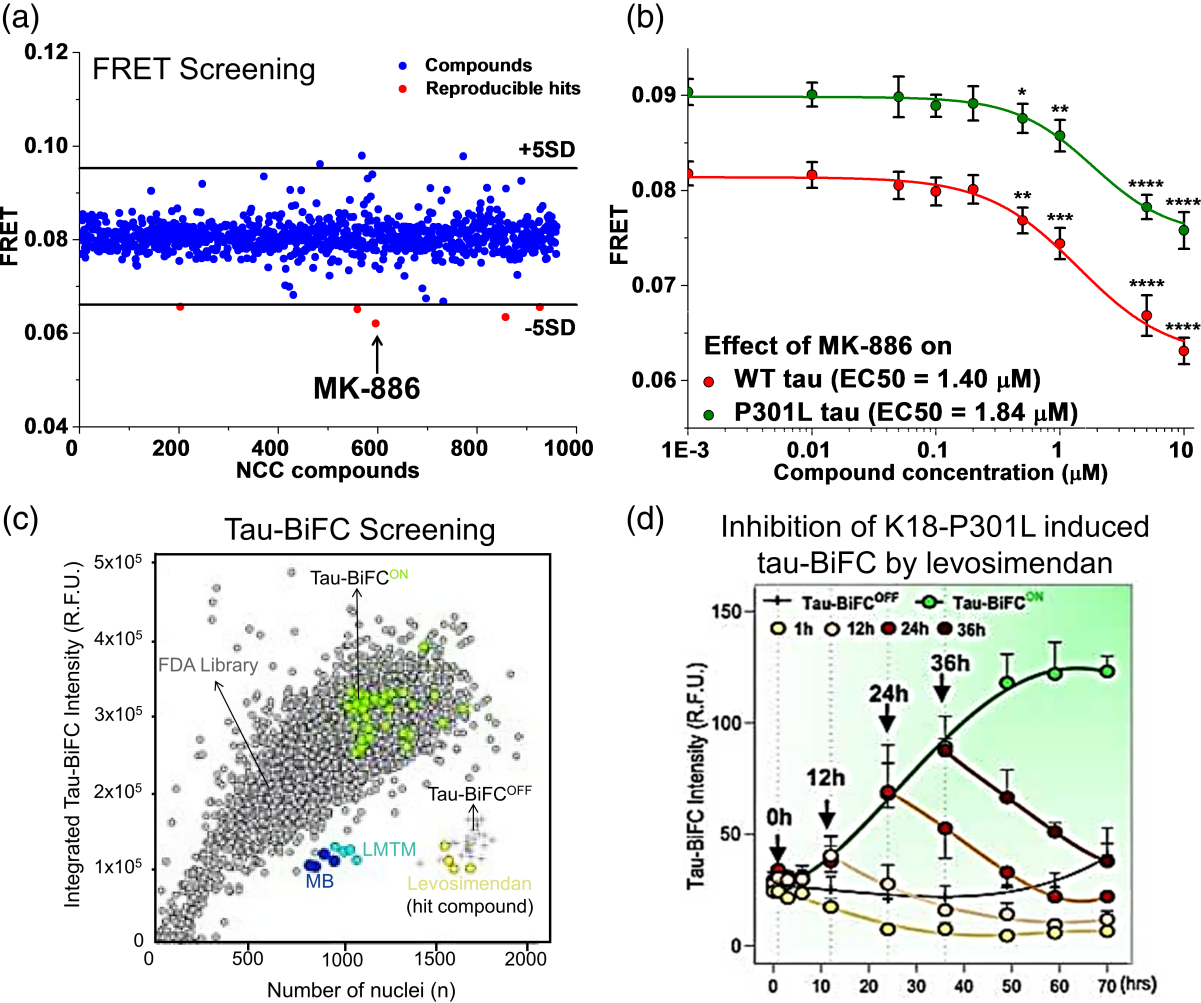
Translational applications of fluorescence resonance energy transfer (FRET) and tau‐bimolecular fluorescence complementation (BiFC) biosensors in high‐throughput screening drug discovery. (a) High‐throughput screening of NIH Clinical Collection (NCC) library containing 727 compounds using wild‐type (WT) 2N4R tau intermolecular FRET biosensor expressed in human embryonic kidney 293 (HEK293) cells to obtain a small molecule inhibitor (MK‐886) of tau oligomerization.^58^ (b) MK‐886 reduces FRET in both 2N4R WT and P301L tau intermolecular biosensors with EC50 values of 1.40 and 1.84 μM, respectively.^58^ (c) High‐throughput screening of 1018 FDA approved compounds using Venus‐based tau‐BiFC fluorescence turn‐on biosensor expressed in HEK293 cells. Tau‐BiFC^OFF^ (+), Tau‐BiFC^ON^ (green), FDA library (gray), methylene blue (MB, blue), LMTM (cyan), and levosimendan (hit compound, yellow) are indicated on the plot.^111^ (d) Treatment of levosimendan at different timings along the aggregation cascade of K18‐P301L induced tau aggregation in tau‐BiFC cells. Inhibitory effect of levosimendan is characterized by decreases in the tau‐BiFC responses.^111^ Permissions obtained from References 58 and 111

We have developed an HTS platform to discover small molecules that target tau oligomerization by combining fluorescent tau biosensor engineering and FRET measurements through high precision and high‐throughput fluorescence lifetime detection.^58^, ^100^ We used WT 2N4R tau FRET biosensor expressed in HEK293 cells to perform screening of NIH Clinical Collection (NCC) library comprising of 727 compounds and identified a novel small molecule, MK‐886, which reduces FRET in the intermolecular tau biosensor (Figure 7(a)).^58^ As HEK293 cells do not express endogenous tau and they have been shown to have good signal‐to‐noise ratio in lifetime‐based FRET studies,^101^, ^102^, ^103^, ^104^, ^105^ they are suitable to be used in HTS to identify modulators of tau oligomer conformations. MK‐886 reduces FRET in a dose‐dependent manner in both WT and tauP301L FRET biosensor expressed in HEK293 cells (Figure 7(b)), and rescues tauP301L‐induced cytotoxicity in SH‐SY5Y cells.^58^ Interestingly, MK‐886 does not fully abolish FRET, suggesting that it may be acting through conversion of toxic tau oligomers into nontoxic oligomers rather than direct disruption of the toxic oligomers, although it remains to be investigated if these nontoxic oligomers are capable of forming neuroprotective fibrils in cells. Using purified protein approach, we show that MK‐886 directly binds tau protein, stabilizes an on‐pathway oligomer and increases the formation of β‐sheet species by altering the conformation of tau monomer at the proline‐rich and microtubule binding regions, as characterized by surface plasmon resonance, ThT and single‐molecule FRET.^58^ In sum, our new tau FRET biosensor and the fluorescence lifetime detection technology are well suited to identify novel compounds capable of remodeling heterogeneous tau oligomers and rescuing tau induced cytotoxicity, thus enabling therapeutic targeting of early‐stage tau pathology. This HTS strategy has also been validated in other systems with α‐synuclein^106^ and huntingtin exon 1,^107^ indicating its robustness in investigating intrinsically disordered proteins.

In a separate study, the above‐mentioned WT 2N4R tau intermolecular FRET biosensor was adopted and expressed in SH‐SY5Y cells to perform HTS on ChemBridge DIVERSet library of 10,000 compounds to identify 195 small molecules that inhibit tau oligomerization.^108^ This small subset of compounds was then filtered by their ability to inhibit purified proteins of α‐synuclein aggregation revealed by ThT assay and dynamic light scattering measurements. MG‐2119 is then identified as a dual mode inhibitor that targets tau oligomerization and α‐synuclein aggregation. MG‐2119 binds tau monomers with high affinity and is able to rescue cell cytotoxicity induced by a combination of tau and α‐synuclein oligomers in SH‐SY5Y cells in a dose‐dependent manner.^108^ In addition, the intermolecular tau FRET biosensor was also used to further filter hits from both acetylcholinesterase inhibitor screen^109^ and microRNA‐146a inhibitor screen^110^ to obtain compounds that serve dual functions, including inhibition of tau oligomerization. These newly identified small molecules and MK‐886 are capable of rescuing cell cytotoxicity induced by different systems, including a combination of tau, α‐synuclein and β‐amyloid oligomers as well as cells co‐expressing tau and miRNA‐146a. It is important to note that some of the compounds that serve dual functions rescue cell cytotoxicity beyond controls, suggesting the promotion of inherent cell growth in addition to inhibiting protein‐induced toxicity. Furthermore, while these compounds are novel and could be effective, it is worth noting that they may potentially contain promiscuous characteristics and exhibit nonspecificity by targeting both tau and other proteins. Hence, the exact inhibition mechanism and mode of action of the compounds require further investigation.

#### 5.2.2 WT 2N4R tau‐BiFC biosensor

The Venus‐based tau‐BiFC biosensor was used to screen a FDA‐approved drug library containing 1018 compounds, and identified levosimendan as a potent small molecule inhibitor of tau oligomerization (Figure 7(c)).^111^ Levosimendan directly inhibits disulfide‐linked tau oligomerization by covalently binding to tau cysteines as characterized by ^14^C‐isotope labeling of the small molecule. In addition, levosimendan prevents the formation of oligomers from monomers and is also capable of disassembling preformed tau oligomers or aggregates into monomers (Figure 7(d)), thus rescuing neurons from tau‐induced toxicity.^111^ The study also made a comparison between levosimendan and the well‐known inhibitors of tau aggregation, methylene blue (MB) and LMTM, and they show that MB and LMTM disrupt fibrils but generate high‐molecular‐weight tau oligomers which are toxic to neurons. A key strength of this study is that they tested levosimendan in vivo with the tauP301L‐BiFC transgenic mice. The administration of levosimendan reduces tau oligomerization as illustrated by a decrease in tau‐BiFC signals, suppresses tau pathology in the brain, and prevents cognitive declines in the mice.^111^

Several tau targeted compounds have been identified from recent drug discovery campaigns which could potentially be further tested and developed into more effective drugs targeting AD and related tauopathies. While HTS assays performed with living cells are able to preserve the native environment as well as capture indirect mechanisms in inhibiting tau oligomerization, it also increases nonspecificity as many other biological processes can be altered in cells that lead to reduction of tau aggregation. It is therefore important to elucidate the binding sites and inhibitory profiles of all compounds identified from HTS to ensure that they are only restrictive to tau‐specific targeting.

## 6 COMPARISON BETWEEN DIFFERENT BIOSENSOR SYSTEMS

We have described three major techniques, FRET, BiFC, and SLC, as well as multiple forms of cellular tau biosensors engineered based on each technique. Fluorescence and bioluminescence methods to quantify tau–tau interactions and conformational dynamics represent promising approaches to probe first‐hand molecular insights of tau aggregation cascade and cell‐to‐cell spreading of tau pathology.^112^, ^113^, ^114^ However, they differ in terms of the type of interactions they can detect, sensitivity and signal‐to‐noise ratio, the possibility of false‐positive or false‐negative signals, capability of spatiotemporal monitoring of interactions, and the instrumentation needed.^77^, ^115^, ^116^ We have summarized the advantages and limitations of each technique (Table 2). In general, expressing these tau biosensors in living cells have the key advantage of preserving the native surroundings, such as the presence of molecular chaperones or other molecular constituents, in which the interaction takes place and is monitored. It has been shown that fluorophore tagging could prevent tau toxicity and nontagged tau should be used for toxicity studies. This also suggests that a common limitation of these biosensors is that they are not capable of monitoring tau‐induced toxicity in cells by themselves, although treatment of exogenous tau oligomers or aggregates to the biosensors has been shown to induce cell cytotoxicity.^99^ In the technical aspects, all three types of biosensors can be used for time‐course analysis and HTS assays, and they are sensitive to changes in local environment.

**TABLE 2:** Advantages and limitations of tau biosensor engineering techniques

Methods	Advantages	Disadvantages/limitations
FRET	Real‐time PPI monitoring as the complexes are in principle at equilibrium which allows for detection of complex formation and dissociation Ability to measure the affinity between protein interactions Ability to detect protein conformational changes, in addition to protein interactions Highly sensitive and can be performed in single cells and single molecules Ability to resolve the molar ratios and the distance distributions between different interacting species in time‐resolved FRET Imaging microscopy‐based FRET detects localization of PPIs while lifetime/intensity‐based FRET provides a high signal‐to‐noise ratio	Requires the attachment of two intrinsic dyes (donor and acceptor) with the same luminousness and stoichiometry Large fluorophores may potentially cause steric hindrance and alter binding properties of interacting partners Weak interacting fluorescent species (e.g., dimerization) may result in inaccuracy of FRET measurements Potential problems with photobleaching or direct excitation of acceptor fluorophore Insensitive to distance outside the dynamic range of the detection of the FRET pair (<10 nm) Low signal‐to‐noise ratio associated with imaging microscopy‐based FRET approach FRET method provides less sensitive analysis of protein interactions as small fractions of interacting proteins combine with each other at any time
BiFC	No requirement for large quantities of proteins, stoichiometric proportions or structural information Proteins with weak interactions or low expressions are able to form complexes in BiFC as there are detectable fluorescence signals even at low level of expressions Noninteracting proteins or endogenous proteins consist of little background fluorescence, resulting in a high signal‐to‐noise ratio The power of the fluorescent signal is directly proportional to the extent of protein interactions Detection of spatiotemporal interaction between two proteins in the living cells The multicolor BiFC assay allows concurrent imaging of several protein complexes within the same cell	The fluorescent intensity of restructured FP fragments should be similar to that of the whole original FP and with adequate brightness Intrinsic interactions or spontaneous assembly between the FP fragments may affect the binding properties of the interacting partners and result in false‐positive signals Difficult to find a suitable negative control while examining the interaction between two new proteins with unknown structural or biochemical information Irreversible BiFC formation results in inability to measure dissociation of protein complexes Slow maturation process affects the visualization of short‐lived interactions in cells Lack of stability may lead to inaccurate measurements
SLC	SLC assays generate low background signals because they do not require external light. Oxidation of chemical substrate by the luciferase emits detectable light known as bioluminescence SLC assays possess a binary on‐or‐off characteristic that increases the detection sensitivity SLC assays can detect both association and dissociation of protein pairs Kinetic measurements of PPI are permitted Large and linear dynamic range of seven to eight orders of magnitude Relatively rapid turnover of the enzyme allows for detection of high luciferase activity	Requirement for exogenous substrates to generate bioluminescence and potential false‐negative results in the presence of a luciferase inhibitor Unlike stable fluorescent signals, the bioluminescence signals in SLC assays gradually decrease because the luciferase–luciferin reaction is a chemical reaction There is a time delay of 4–6 h from stimulus to response to allow transcription to occur SLC assay is not suitable for monitoring PPI over a long time (>5 h) Unable to measure physical distances between a protein pair as the minimum physical distance required to obtain an interaction signal in SLC is unknown

Abbreviations: BiFC, bimolecular fluorescence complementation; FP, fluorescent protein; FRET, fluorescence resonance energy transfer; GSK3β, glycogen synthase kinase 3 beta; PPIs, protein–protein interactions; SLC, split luciferase complementation.

### 6.1 Bimolecular fluorescence complementation

The BiFC approach allows for tagging of smaller fluorophores, a low background fluorescence as noise and signal detection even at low level of protein expression.^117^, ^118^ The multicolor BiFC assay allows concurrent imaging of several protein complexes within the same cell such as tau–tau interactions and interactions between tau and other proteins which might change tau aggregation propensity.^117^ However, the process of fluorophore fusion is not reversible. Once a transient complex between the protein of interest is formed, the reconstituted fluorescence protein remains relatively stable, hence reducing its capability in continuously monitoring the tau aggregation and disaggregation process.^118^ In addition, the split fluorescent protein might autonomously and spontaneously assemble, which will alter the binding properties of interacting tau and increase the false‐positive signals.^115^, ^117^

### 6.2 Split luciferase complementation

A key advantage of SLC assays is the low background signals as they do not require external light for detection and allows a binary on‐or‐off characteristic that increases the detection sensitivity.^119^, ^120^ It also has a large and linear dynamic range with seven to eight orders of magnitude.^121^ However, the presence of a luciferase inhibitor may generate false‐negative results.^122^ In addition, it is unable to measure distance between proteins and is not suitable for monitoring protein interactions over a long time as the bioluminescence signals gradually decrease over time.^119^

### 6.3 Fluorescence resonance energy transfer

FRET is powerful in determining spatiotemporal dynamics and reversibility of PPIs instantaneously at real time.^54^, ^118^ Besides measuring direct tau–tau intermolecular interactions, it also allows measurements of tau intramolecular folding or conformational change.^58^ However, it requires large fluorophore fusion which may cause steric hindrance.^123^ In addition, it has disadvantages of irreversible photobleaching, being less sensitive due to small fractions of interacting proteins combine with each other at any time, and insensitive to distance outside the dynamic range of the detection of the FRET pair.^124^, ^125^ Despite these limitations, important recent advances in lifetime‐based FRET measurements allow for data fitting of the acquired fluorescent waveforms with sufficient precision to analyze two or more samples having different lifetimes, and resolving multiple components with high accuracy with respect to both lifetime and mole fraction.^126^, ^127^

The potential of resolving the different populations of protein species and the distance distributions of PPIs through model fitting of the time‐resolved FRET waveforms containing high‐content information^127^ can be in theory applied to the investigation of tau oligomers and aggregates. However, a major limitation of tau oligomer associated time‐resolved FRET data lies in the lack of information such as the number of interacting tau monomers, the stoichiometry of the tau oligomer or well‐defined structural states required to constrain the model in order to identify specific toxic species. This suggests the need to perform additional complementary biophysical and spectroscopic characterizations such as analytical ultracentrifugation, nuclear magnetic resonance, electron paramagnetic resonance, surface plasmon resonance, or Raman spectroscopy to obtain oligomer size and binding affinity, although these techniques are mostly conducted with purified proteins in current studies and may not be directly correlated to cellular observations.

## 7 CONCLUSION AND FUTURE PERSPECTIVES

The advancements in the cellular biosensor technology allow for the studies of molecular mechanisms involved in tau oligomerization and aggregation, as well as in cell‐to‐cell spreading of tau pathology. The tau biosensors can further be used to study the effect of different tau isoforms, mutations, and posttranslational modifications on the formation of toxic tau oligomers. It might be important to perform cross‐validation experiments with different biosensors to ensure an observation or finding is consistent and reproducible with different tools. It is also imperative to compare biophysical observations from cellular models to tau oligomers extracted from AD mouse models or patients to elucidate the structural states of the true toxic tau oligomeric species. The use of FRET, BiFC and SLC assays can also be used to study cross reactivity between tau and other intrinsically disordered proteins such as β‐amyloid^128^ and α‐synuclein.^129^ To improve on the physiological relevance, the tau biosensors can be expressed in human induced pluripotent stem cells. Furthermore, future drug discovery campaigns using tau biosensors should include screening of CNS‐focused compound libraries to ensure tau‐targeted small molecules have a high probability of crossing the blood–brain barrier for more effective treatment of AD and related tauopathies.

## CONFLICT OF INTEREST

The author declares no conflict of interest.

## AUTHOR CONTRIBUTIONS

**Chih Hung Lo**: Conceived the idea and wrote the manuscript.

## Data Availability

Data sharing not applicable to this article as no datasets were generated or analyzed during the current study.
